# Efficacy of omeprazole, famotidine, mosapride and teprenone in patients with upper gastrointestinal symptoms: an omeprazole-controlled randomized study (J-FOCUS)

**DOI:** 10.1186/1471-230X-12-42

**Published:** 2012-05-01

**Authors:** Kouichi Sakurai, Akihito Nagahara, Junichi Akiyama, Junichi Suzuki, Yasuki Habu, Akihiro Araki, Tsuyoshi Suzuki, Katsuaki Satoh, Haruhiko Nagami, Ryosaku Harada, Nobuo Tano, Masayasu Kusaka, Yasuhiko Fujioka, Toshikatsu Fujimura, Nobuyuki Shigeto, Tsuneyo Oumi, Jun Miwa, Hiroto Miwa, Kazuma Fujimoto, Yoshikazu Kinoshita, Ken Haruma

**Affiliations:** 1 Department of Gastroenterology and Hepatology, Graduate School of Medical Sciences, Kumamoto University, 1-1-1 Honjo, Kumamoto, 860-8556, Japan; 2 Department of Gastroenterology, Juntendo University School of Medicine, Tokyo, Japan; 3 Internal Medicine, Matsue Red Cross Hospital, Matsue, Japan; 4 Department of Gastroenterology, International Medical Centre, Tokyo, Japan; 5 Department of Internal Medicine, Yamagata Prefectural Central Hospital, Yamagata, Japan; 6 Department of Gastroenterology, KKR Sapporo Medical Center, Sapporo, Japan; 7 Department of Gastroenterology, Saiseikai Noe Hospital, Osaka, Japan; 8 Department of Gastroenterology and Hepatology, Graduate School of Medical and Dental Sciences, Tokyo Medical and Dental University, Tokyo, Japan; 9 Department of Gastroenterology, Tokyo Metropolitan Police Hospital, Tokyo, Japan; 10 Satoh Gastrointestinal Surgical Hospital, Kurashiki, Japan; 11 Nagami Clinic, Unnan, Japan; 12 Gastrointestinal Division, Harada Internal Medicine, Tosu, Japan; 13 Department of Internal Medicine, Kyoritsu Hospital, Kawanishi, Japan; 14 Kusaka Hospital, Bizen, Japan; 15 Fujioka Hospital, Saga, Japan; 16 Yamaguchi Hospital, Imari, Japan; 17 Department of Internal Medicine, Tamano City Hospital, Tamano, Japan; 18 Ooumi Clinic, Tsuyama, Japan; 19 Department of Internal Medicine, Toshiba Hospital, Tokyo, Japan; 20 Department of Internal Medicine, Hyogo College of Medicine, Nishinomiya, Japan; 21 Department of Internal Medicine, Gastrointestinal Endoscopy, Saga Medical School, Saga, Japan; 22 Department of Gastroenterology and Hepatology, Shimane University School of Medicine, Izumo, Japan; 23 Division of Gastroenterology, Department of Internal Medicine, Kawasaki Medical School, Kurashiki, Japan; 24 Department of General Medicine, Kawasaki Medical School, Kurashiki, Japan; 25 Department of Gastroenterology, Hokkaido University, Sapporo, Japan

**Keywords:** Omeprazole, Famotidine, Mosapride, Teprenone, Uninvestigated upper gastrointestinal symptoms

## Abstract

**Background:**

In Japan, treatment guidelines are lacking for patients with upper gastrointestinal symptoms. We aimed to compare the efficacy of different drugs for the treatment of uninvestigated upper gastrointestinal symptoms.

**Methods:**

This was a randomized, open-label, parallel-group multicenter study. *Helicobacter pylori*-negative, endoscopically uninvestigated patients ≥ 20 years of age with upper gastrointestinal symptoms of at least moderate severity (Global Overall Symptom score [GOS] ≥ 4 on a 7-point Likert scale) were randomized to treatment with omeprazole (10 mg once daily), famotidine (10 mg twice daily), mosapride (5 mg three times daily) or teprenone (50 mg three times daily). The primary endpoint was sufficient relief of upper gastrointestinal symptoms after 4 weeks of treatment (GOS ≤ 2). UMIN clinical trial registration number: UMIN000005399.

**Results:**

Of 471 randomized patients, 454 were included in the full analysis set. After 4 weeks of treatment, sufficient symptom relief was achieved by 66.9% of patients in the omeprazole group, compared with 41.0%, 36.3% and 32.3% in the famotidine, mosapride and teprenone groups, respectively (all, p < 0.001 vs omeprazole). There were no treatment-related adverse events.

**Conclusions:**

The favorable efficacy and safety profiles of omeprazole in relieving uninvestigated upper gastrointestinal symptoms support its use as first-line treatment in this patient group in Japan. Patients who show no improvement in symptoms despite PPI use, and those with alarm symptoms (such as vomiting, GI bleeding or acute weight loss) should receive further investigation, including prompt referral for endoscopy.

**Trial registration:**

UMIN000005399.

## Background

Upper gastrointestinal (GI) symptoms are common in Japan and their prevalence may be increasing. In a large, international population-based survey of 500 Japanese participants, 26% had experienced upper GI symptoms during the previous 3 months and had a reduced quality of life as a consequence [1,2]. Similarly, in a survey of 231 Japanese employees, 20% reported having frequent upper GI symptoms in the previous 3 months [3]. An international review of the prevalence of gastroesophageal reflux disease (GERD) showed that, although the prevalence of heartburn and/or regurgitation on at least 2 days per week was lower in Japan (about 7%) than in Western populations, it was increasing [4].

*Helicobacter pylori* infection is a common cause of underlying peptic ulcer disease and might be a cause of upper GI symptoms [5]. Hence, a *H. pylori* ‘test and treat’ strategy is the mainstay for managing patients with uninvestigated upper GI symptoms [6,7]. Another cause of upper GI symptoms is GERD [8]. The presence of upper GI symptoms in the absence of GERD or organic findings at endoscopy is termed functional dyspepsia. The Rome III classification of functional GI disorders lists symptoms of postprandial fullness, early satiety, epigastric pain and epigastric burning as diagnostic criteria for functional dyspepsia when there is no evidence of structural disease that is likely to explain the symptoms [9]. Bloating, postprandial nausea and excessive belching can also support a diagnosis of functional dyspepsia [9]. By contrast, the predominant symptoms of heartburn and/or regurgitation are excluded from the criteria for dyspepsia and are instead included in the diagnostic criteria for GERD, with or without reflux esophagitis [8,9]. However, patients with upper GI diseases/disorders often present with multiple symptoms, and symptoms corresponding to GERD and dyspepsia frequently coexist in the same patient [10,11]. For example, in a Japanese study by Adachi *et al.* of 221 patients with GERD who had reflux esophagitis, the mean number of upper GI symptoms reported by each patient was 5.4 [10]. Similarly, in the Canadian Adult Dyspepsia Empirical Treatment–Prompt Endoscopy (CADET-PE) study by Thomson *et al.*, of 1040 patients with uninvestigated dyspeptic symptoms, 80% had at least six upper GI symptoms, including heartburn, regurgitation, upper abdominal pain and upper abdominal fullness, while < 0.1% had only one symptom [11].

Acid suppression with proton pump inhibitors (PPIs) is effective in relieving heartburn and regurgitation, and the American College of Gastroenterology guidelines recommend PPIs as the mainstay therapy for GERD [12]. PPIs are also recommended as first-line pharmacotherapy for the management of dyspepsia [13,14], and are more effective than histamine H_2_-receptor antagonists or prokinetic agents in the treatment of upper GI symptoms in patients with *H. pylori*-negative dyspepsia [15]. There is, however, a need for up-to-date management guidelines for GERD (with and without reflux esophagitis) and dyspepsia in Japan [16,17].

Studies on the efficacy of PPIs for upper GI symptoms are lacking for Japanese populations, which have a lower prevalence of heartburn and regurgitation than Western populations [1,2,4]. Therefore, in an effort to rectify this situation, we conducted a study of four GI medications, the omeprazole-controlled randomized study in Japanese patients with upper GI symptoms (J-FOCUS). The study included endoscopically uninvestigated, *H. pylori*-negative patients with upper GI symptoms. The aim of J-FOCUS was to compare the symptomatic efficacy of the PPI omeprazole with that of three other GI drugs with different modes of action: the histamine H_2_-receptor antagonist famotidine, the prokinetic agent mosapride and the mucosal protective agent teprenone.

## Methods

### Study design

This randomized, multicenter, parallel-group, open-label study was conducted in Japan between February 2007 and June 2008. The local ethics committee at each study center approved the study protocol. We obtained written informed consent from all of the patients.

### Setting and participants

J-FOCUS was conducted at 162 centers in Japan. The study enrolled patients who visited a participating clinic or hospital because of GI symptoms. Patients aged ≥ 20 years of age were eligible for inclusion if they had chronic or recurrent episodes of at least one of eight specific upper GI symptoms (epigastric pain, heartburn, regurgitation, postprandial fullness, nausea/vomiting, belching, early satiety and/or bloating), with one or more of the symptoms being of at least moderate severity (Global Overall Symptom [GOS] score ≥ 4) in the previous week. All patients underwent urine antibody testing (Rapirun® *H. pylori* Antibody Detection Kit, Otsuka Pharmaceutical Co., Ltd., Tokyo, Japan). This test was reported to show high sensitivity (100%) and accuracy (95.2%) for diagnosis of *H. pylori* infection relative to biopsy-based testing [18]. Individuals who were *H. pylori* positive, requiring *H. pylori* eradication therapy, were not included in this study. Patients with a history of *H. pylori* eradication were excluded because this could introduce errors with antibody testing. Patients were also excluded if they had undergone an endoscopy in the previous 3 months; had alarm symptoms (such as vomiting, GI bleeding or acute weight loss) requiring endoscopy; were judged by the investigator to require a prompt endoscopy; had a confirmed or suspected malignant lesion; had prior GI resectioning or vagotomy; had irritable bowel syndrome or other comorbidities (including hepatic, renal or cardiac disease); had severe mental illness; were or might possibly be pregnant or were lactating; or were judged to be ineligible for study entry by the investigator.

PPIs, H_2_-receptor antagonists, prokinetic agents, gastric mucosal protective agents, anticholinergics, antidepressants, anxiolytics, steroids (other than topical steroids), non-steroidal anti-inflammatory drugs, aspirin or bisphosphonates were discontinued at least 1 week before study entry and were not allowed during the study period.

### Randomization and interventions

Eligible patients were randomly assigned in a 2:2:2:1 proportion, using a central computer-generated randomization list managed by a clinical research coordinator at each center. During screening/enrollment, the physician recorded the subject’s characteristics and provided this information to the clinical research coordinator, who then allocated the subject an ID and study drug based on the sealed allocation tables prepared by the secretariat. Patients were allocated using this method to receive omeprazole (10 mg once daily), famotidine (10 mg twice daily), mosapride (5 mg three times daily) or teprenone (50 mg three times daily) for 4 weeks. All of the drugs were prescribed routinely and administered orally. The doses of each drug were in line with the authorized doses that are considered optimal for the treatment of dyspepsia or GERD symptoms in Japan. Rescue medication was not allowed. Patients visited the clinic at study entry and at 4 weeks after the start of treatment, and completed the GOS assessment. An optional additional clinic visit could take place at 2 weeks after the start of treatment. There were no deviations in the allocation of subjects based on their background characteristics.

### Outcomes and follow-up

The primary endpoint was the proportion of patients with sufficient overall symptom relief after 4 weeks of treatment, which was defined as, at most, minimal symptom severity (GOS ≤ 2) for all symptoms on the GOS. The GOS has been validated in patients with dyspepsia [19], and has been used in clinical studies of patients with dyspepsia to assess symptoms and treatment success [15,20,21]. It measures the severity of eight symptoms (epigastric pain, heartburn, regurgitation, postprandial fullness, nausea/vomiting, belching, early satiety and bloating) on a 7-point Likert scale (1 = no problem [no symptoms]; 2 = minimal problem [can be easily ignored without effort]; 3 = mild problem [can be ignored with effort]; 4 = moderate problem [cannot be ignored but does not influence daily activities]; 5 = moderately severe problem [cannot be ignored and occasionally limits daily activities]; 6 = severe problem [cannot be ignored and often limits concentration on daily activities]; 7 = very severe problem [cannot be ignored, markedly limits daily activities and often requires rest]. The completed GOS was collected by the investigators who were not allowed to change any outcome reported by the patients.

Secondary endpoints were the proportion of patients with complete overall symptom relief (GOS = 1) after 4 weeks of treatment for all symptoms on the GOS; the proportion of patients with sufficient overall symptom relief after 2 weeks of treatment; the proportion of patients with complete overall symptom relief after 2 weeks of treatment; the proportion of patients with symptom improvement after 4 weeks of treatment (decrease in GOS by ≥ 2 grades for each symptom scored ≥ 3 at baseline on the GOS); and the proportion of patients with symptom aggravation (increase in GOS by ≥ 2 grades for each symptom scored ≤ 5 at baseline on the GOS) after 4 weeks of treatment.

Upper GI symptoms were classified into one of the following three categories: (1) GERD symptoms, if the symptom with the highest GOS score was heartburn or regurgitation; (2) dyspeptic symptoms, if the symptom with the highest GOS score was epigastric pain, postprandial fullness or early satiety; (3) and other upper GI symptoms, if the symptom with the highest GOS score was nausea, vomiting, belching or bloating. Coexistence was defined as the presence of symptoms from more than one symptom category with a score of ≥ 4. Treatment-related adverse events were assessed and recorded at each follow-up visit.

### Statistical analysis

Based on prior studies [10,15], we estimated the proportion of patients with symptom relief after 4 weeks of treatment at 68% for omeprazole and 50% for famotidine. From these values, we calculated that 127 patients in each of the omeprazole and famotidine treatment groups would provide 80% power to detect a significant difference between the two groups. We aimed for a similar number of patients for the mosapride group. The effect of teprenone on dyspeptic or GERD symptoms was assumed to be lower than that of the other agents [22]. Considering the symptom severity of included patients, it was planned for ethical reasons to keep the group allocated to teprenone as small as possible. It was estimated that the effect of teprenone could be confirmed with half the number of individuals allocated to the other agents.

Safety variables were analyzed using all randomized patients (i.e., intention-to-treat population). Efficacy variables were analyzed using the full analysis set, which comprised all patients who attended the baseline visit and at least one follow-up visit. Patients who did not return after the initial visit were excluded from efficacy analyses. The primary variable, the proportion of patients with sufficient overall symptom relief after 4 weeks of treatment, was compared between the omeprazole group and the other treatment groups using *χ*^2^ tests. A value of p < 0.0167 in a two-sided test with Bonferroni correction was considered statistically significant. For secondary variables, no corrections were made for multiple comparisons. Multivariate logistic regression analysis was also conducted to examine potential determinants of treatment efficacy.

## Results

### Study sample

**Figure 1:**
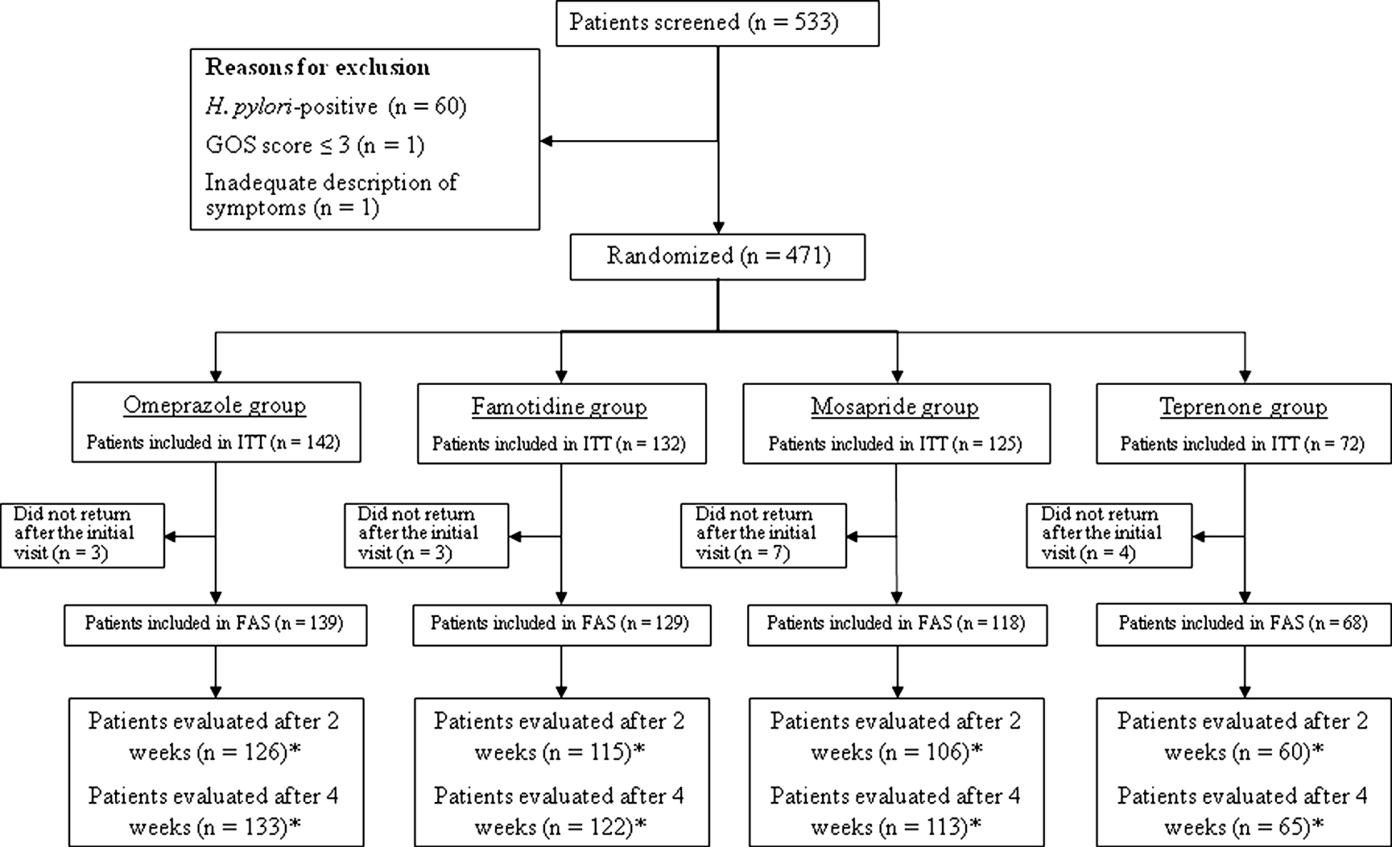
**Study flow diagram.** *patients who completed the Global Overall Symptom assessment; ITT, intention-to-treat; FAS, full analysis set.

In total, of 533 patients with upper GI symptoms who were screened, 62 were excluded (*H. pylori* positive, n = 60; GOS ≤ 3, n = 1; incomplete questionnaire, n = 1) and 17 were lost to follow-up after randomization. Thus, 471 patients were included in the intention-to-treat analysis and 454 patients were included in the full analysis set (Figure 1). In the 1 month before study entry, 83% of patients were not prescribed a GI drug: no patient was prescribed a PPI or H_2_-receptor antagonist, 5% were prescribed a prokinetic and 12% were prescribed other GI drugs. The characteristics of the patients at baseline were similar in the four treatment groups (Table 1).

**Table 1:** Patient characteristics at baseline

	**Omeprazole group (n = 139)**		**Famotidine group (n = 129)**		**Mosapride group (n = 118)**		**Teprenone group (n = 68)**	
**Men, n (%)**	60	(43.2%)	48	(37.2%)	40	(33.9%)	29	(42.6%)
**Age y, n (%)**								
≤39	72	(51.8%)	70	(54.3%)	61	(51.7%)	41	(60.3%)
40–59	52	(37.4%)	51	(39.5%)	45	(38.1%)	21	(30.9%)
≥60	15	(10.8%)	8	(6.2%)	12	(10.2%)	6	(8.8%)
**Age, y (mean ± SD)**	40.9 ± 13.6		39.4 ± 12.6		40.0 ± 13.2		40.0 ± 12.9	
**BMI, kg/m**^**2**^								
<20	36	(25.9%)	42	(32.6%)	33	(28.0%)	20	(29.4%)
20–24	79	(56.8%)	65	(50.4%)	64	(54.2%)	39	(57.4%)
≥25	24	(17.3%)	22	(17.1%)	21	(17.8%)	9	(13.2%)
**BMI, kg/m**^**2**^**(mean ± SD)**	22.3 ± 3.5		21.9 ± 3.0		22.0 ± 3.2		21.7 ± 3.0	
**Smoking status (cigarettes/day)**								
None	101	(72.7%)	101	(78.3%)	93	(78.8%)	47	(69.1%)
<20	35	(25.2%)	20	(15.5%)	19	(16.1%)	16	(23.5%)
≥20	3	(2.2%)	8	(6.2%)	6	(5.1%)	5	(7.4%)
**Alcohol intake**								
None	59	(42.4%)	55	(42.6%)	42	(35.6%)	22	(32.4%)
Occasionally	58	(41.7%)	53	(41.1%)	45	(38.1%)	32	(47.1%)
Every day	22	(15.8%)	21	(16.3%)	31	(26.3%)	14	(20.6%)
**Concurrent disease**								
Absent	111	(79.9%)	114	(88.4%)	98	(83.1%)	62	(91.2%)
Present	28	(20.1%)	15	(11.6%)	20	(16.9%)	6	(8.8%)

SD, standard deviation; BMI, body mass index.

### Symptoms at baseline

**Figure 2:**
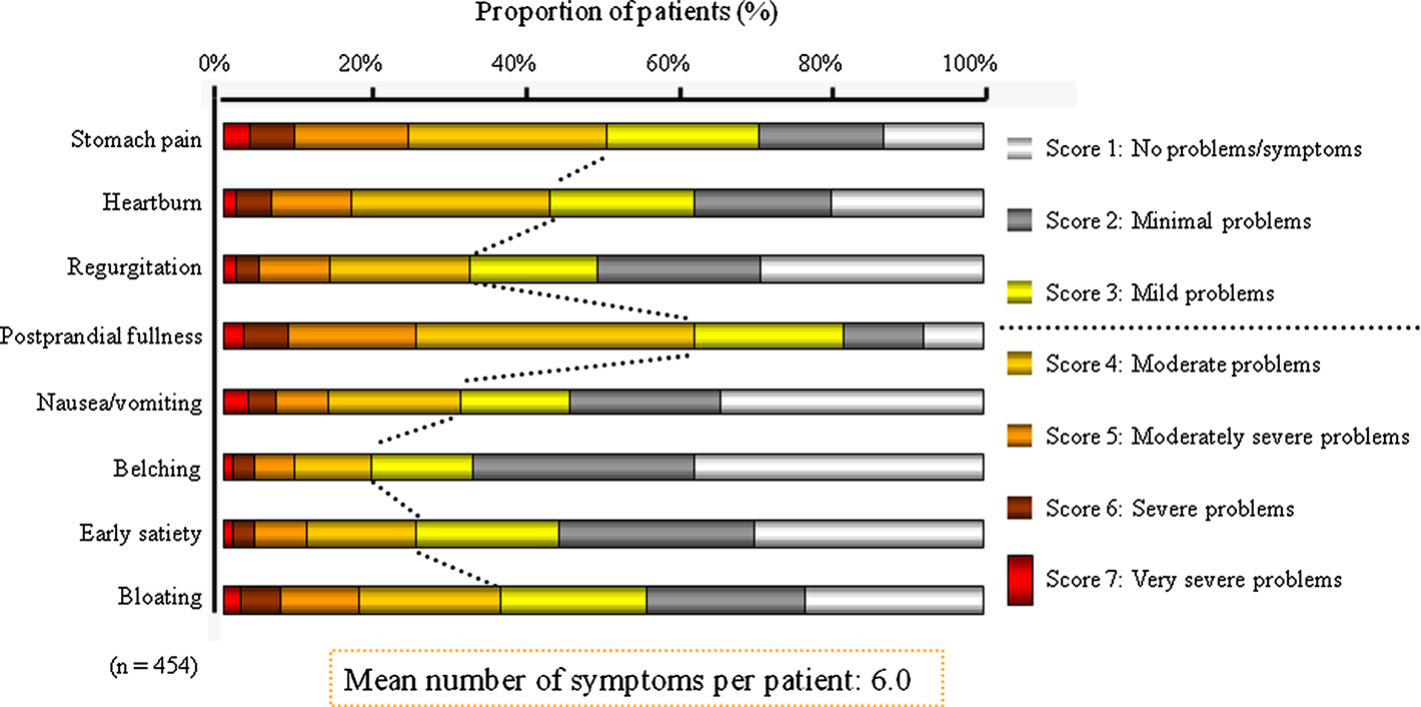
Upper gastrointestinal symptoms in 454 patients at baseline, as scored on the Global Overall Severity (GOS) scale.

**Figure 3:**
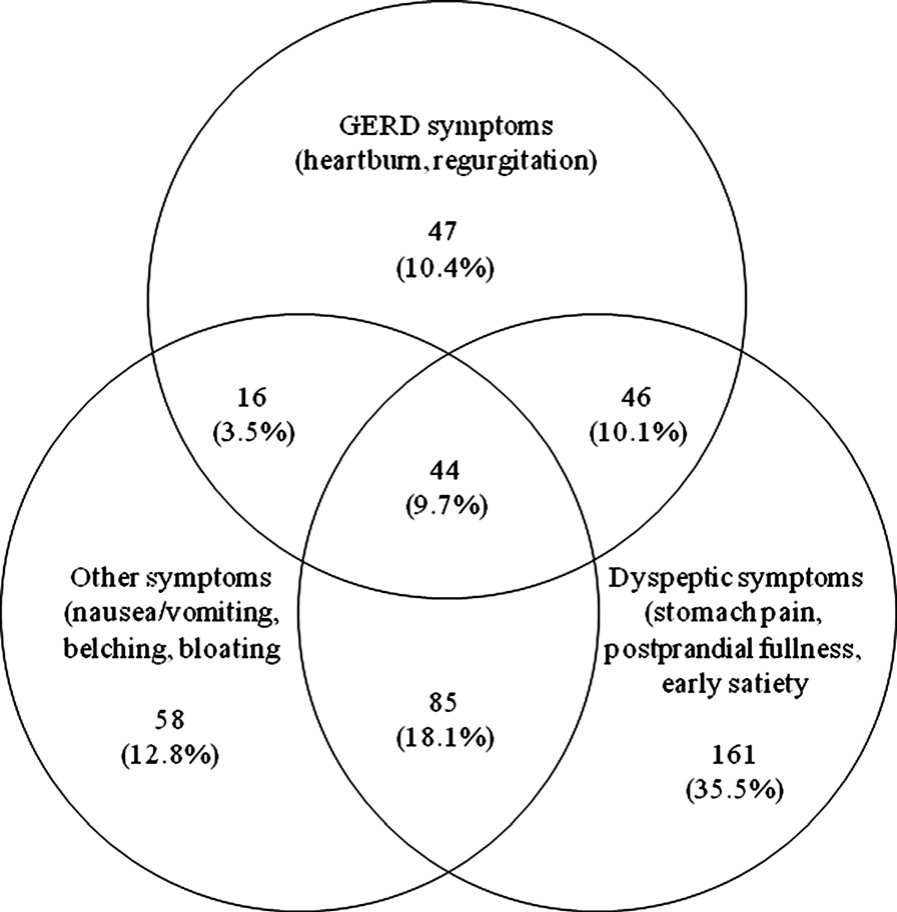
**Coexistence of predominant gastroesophageal reflux disease (GERD) symptoms, predominant dyspeptic symptoms and predominant other upper gastrointestinal symptoms, according to the symptom with the highest Global Overall Severity score at baseline.** A joint highest score for more than one symptom was possible. GERD symptoms included heartburn and regurgitation; dyspeptic symptoms included epigastric pain, postprandial fullness and early satiety; and other symptoms included nausea/vomiting, belching and bloating.

The mean number of upper GI symptoms per patient was 6.0 at baseline. In terms of symptoms of at least moderate severity (i.e. GOS ≥ 4) postprandial fullness was the most frequent, being reported by 69.1% of patients, followed by epigastric pain, heartburn and bloating (Figure 2). Overall, 41.4% of patients had more than one predominant symptom type (‘GERD symptoms’, ‘dyspeptic symptoms’ and ‘other upper GI symptoms’; Figure 3). Ninety patients reported both GERD symptoms and dyspeptic symptoms as their predominant symptom type. There were no significant differences in the proportions of patients with each symptom between the four treatment groups at study entry (Table 2).

**Table 2:** Number of patients with each symptom at baseline

	**Omeprazole group (n = 139)**		**Famotidine group (n = 129)**		**Mosapride group (n = 118)**		**Teprenone group (n = 68)**	
**Stomach pain**	87	(14.6)	90	(17.5)	74	(15.2)	51	(16.1)
**Heartburn**	89	(15.0)	75	(14.6)	64	(13.1)	43	(13.6)
**Regurgitation**	66	(11.1)	54	(10.5)	60	(12.3)	35	(11.1)
**Postprandial fullness**	113	(19.0)	100	(19.5)	87	(17.9)	55	(17.4)
**Vomiting**	59	(9.9)	51	(9.9)	54	(11.1)	29	(9.2)
**Belching**	46	(7.7)	31	(6.0)	34	(7.0)	30	(9.5)
**Early satiety**	60	(10.1)	47	(9.2)	51	(10.5)	33	(10.4)
**Bloating**	75	(12.6)	65	(12.7)	63	(12.9)	40	(12.7)

Values are n (%). There were no significant differences in rates of each symptom across treatment groups (all, p > 0.05).

### Primary outcome

**Figure 4:**
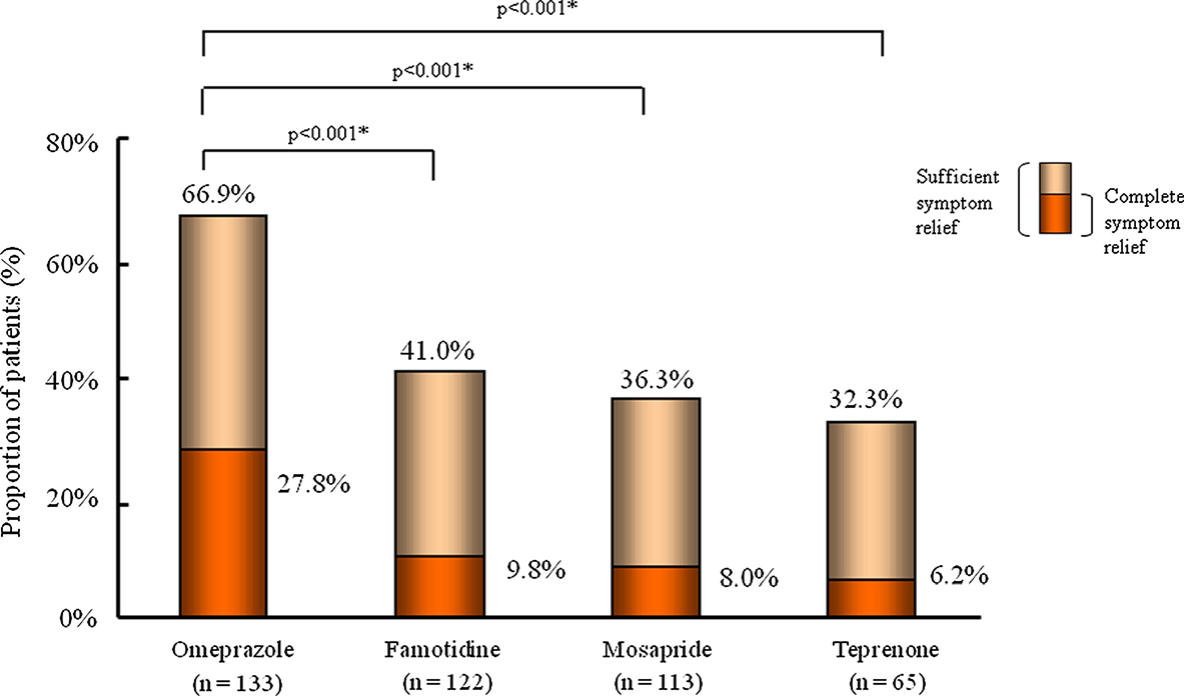
**Proportion of patients with sufficient and complete overall relief of their upper gastrointestinal symptoms after 4 weeks of treatment with omeprazole, famotidine, mosapride or teprenone.** Symptoms were scored using the Global Overall Severity (GOS) scale. Sufficient overall symptom relief was defined as GOS ≤ 2 and complete overall symptom relief was defined as GOS = 1 for all symptoms. *p-values given for sufficient symptom relief.

The proportion of patients with sufficient overall symptom relief after 4 weeks of treatment was significantly higher in the omeprazole group than in the other treatment groups. In total, 66.9% of patients in the omeprazole group reported sufficient overall symptom relief after 4 weeks of treatment, compared with 41.0%, 36.3% and 32.3% in the famotidine, mosapride and teprenone groups, respectively (p < 0.001 vs omeprazole; Figure 4). Similar results were obtained in the intent-to-treat population, consisting of all randomized patients (omeprazole, 62.7%; famotidine, 37.9%; mosapride, 32.8%; and teprenone, 24.2%; all p < 0.001 vs omeprazole).

### Secondary outcomes

The proportion of patients with complete overall symptom relief after 4 weeks of treatment was significantly higher in the omeprazole group than in any of the other three treatment groups. In total, 27.8% of the patients treated with omeprazole reported complete overall symptom relief after 4 weeks of treatment, as compared with 9.8%, 8.0% and 6.2% of patients treated with famotidine, mosapride and teprenone, respectively (all, p < 0.001 vs omeprazole).

Sufficient overall symptom relief after 2 weeks of treatment was reported by 37.3% of patients in the omeprazole group, compared with 20.0% in the famotidine group (*P* = 0.003 vs omeprazole), 15.1% in the mosapride group (p < 0.001 vs omeprazole) and 20.0% in the teprenone group (p = 0.018 vs omeprazole). The proportion of patients with complete overall symptom relief after 2 weeks of treatment was 8.7% in the omeprazole group, compared with 7.0% in the famotidine group (p = 0.610 vs omeprazole), 0.9% in the mosapride group (p = 0.008 vs omeprazole) and 6.7% in the teprenone group (p = 0.629 vs omeprazole).

**Figure 5:**
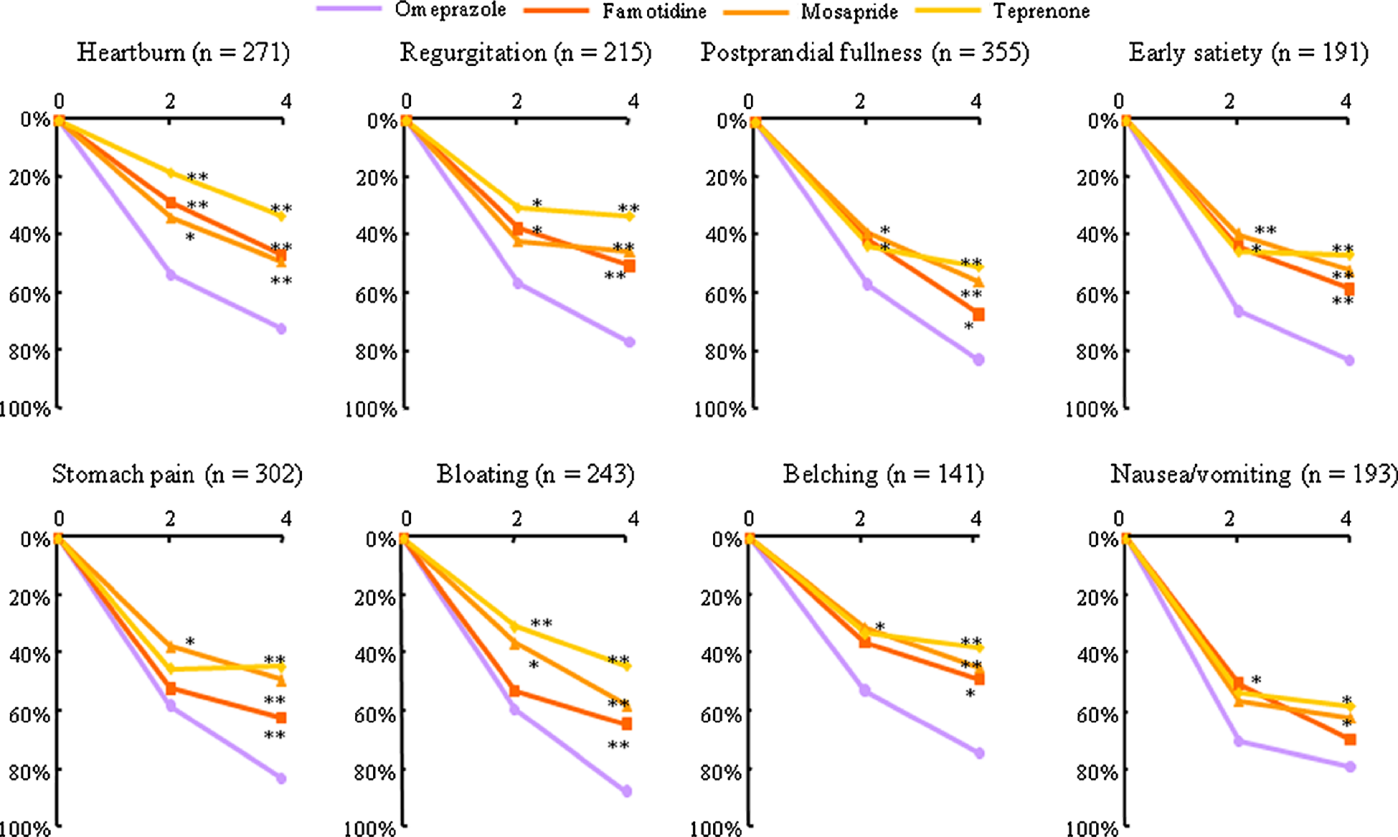
**Proportion of patients with improvements in upper gastrointestinal symptoms after 2 weeks and 4 weeks of treatment with omeprazole, famotidine, mosapride or teprenone.** Symptoms were scored using the Global Overall Severity (GOS) scale, and symptom improvement was defined as a decrease in GOS by ≥ 2 grades. *p < 0.05, **p < 0.01 vs omeprazole.

The largest effect on symptom improvement was observed in the omeprazole group for each of the eight symptoms assessed by the GOS (Figure 5). By contrast, the proportion of patients with symptom aggravation after 4 weeks of treatment was highest in the teprenone group and lowest in the omeprazole group: 1.5% of patients in the omeprazole group reported symptom aggravation after 4 weeks of treatment, compared with 4.9% in the famotidine group (p = 0.118 vs omeprazole), 6.2% in the mosapride group (p = 0.051 vs omeprazole) and 10.8% in the teprenone group (p = 0.003 vs omeprazole).

Compliance was assessed as the proportion of patients who took at least 75% of their doses over the treatment period. The rates of compliance were 96%, 91%, 84% and 89% in the omeprazole, famotidine, mosapride, and teprenone groups, respectively. None of the patients included in the safety analysis set reported any treatment-related adverse events at either follow-up visit.

### Factors affecting outcome

Multivariate logistic regression analysis showed that patients in the famotidine, mosapride and teprenone treatment groups were significantly less likely than those in the omeprazole treatment group to have sufficient overall symptom relief after 4 weeks of treatment (Table 3). Logistic regression analysis for all treatment groups combined showed that sufficient overall symptom relief after 4 weeks of treatment was more likely in patients with fewer than five upper GI symptoms than in those with five or more upper GI symptoms at baseline. Symptom relief was also more likely in patients with predominant dyspepsia symptoms than in those without predominant dyspepsia symptoms at baseline (Table 3).

**Table 3:** Demographic and clinical characteristics and their association with sufficient overall symptom relief after 4 weeks of treatment on multivariate logistic regression analysis

**Variable**	**OR (95% CI)**
**Drug**	
Omeprazole	1.00
Teprenone	0.22 (0.11–0.42)
Mosapride	0.28 (0.16–0.48)
Famotidine	0.30 (0.17–0.51)
**Sex**	
Male	1.00
Female	0.80 (0.49–1.32)
**Age,*y***	
< 60	1.00
≥ 60	1.0 (0.98–1.01)
**BMI,*kg/m***^***2***^	
< 25	1.00
≥ 25	0.98 (0.91–1.06)
**Smoking status**	
Non-smoker	1.00
Smoker	1.01 (0.69–1.48)
**Alcohol intake**	
Non-drinker	1.00
Drinker	0.94 (0.69–1.28)
**Number of symptoms at baseline**	
< 5	1.00
≥ 5	0.48 (0.29–0.81)
**Symptom severity**	
Less than severe	1.00
Severe	0.65 (0.39–1.08)
**Symptom type**	
GERD-type*	1.23 (0.78–2.0)
FD-type*	1.67 (1.01–2.8)
Others*	0.85 (0.55–1.30)

*Compared with patients who did not have this symptom type. OR, odds ratio; CI, confidence interval; BMI, body mass index; FD, functional dyspepsia; GERD, gastroesophageal reflux disease.

The proportion of patients with sufficient overall symptom relief after 4 weeks of treatment was highest in the omeprazole group, regardless of symptom severity, number of symptoms and symptom type at baseline (Table 3). The proportion of patients who reached the primary endpoint in the omeprazole group was higher among those with predominant GERD symptoms (76.8%) than among those with predominant dyspeptic symptoms (68.4%) and those with predominant other upper GI symptoms (62.7%). Other than the omeprazole group, the highest proportion of patients who reached the primary endpoint was found in the famotidine group with predominant dyspeptic symptoms (Table 4).

**Table 4:** Proportion of patients with sufficient overall symptom relief after 4 weeks of treatment with omeprazole, famotidine, mosapride or teprenone, grouped by symptom severity, number of symptoms and symptom type

		**Omeprazole**		**Famotidine**		**Mosapride**		**Teprenone**	
		**n**	**Proportion with sufficient overall symptom relief**	**n**	**Proportion with sufficient overall symptom relief**	**n**	**Proportion with sufficient overall symptom relief**	**n**	**Proportion with sufficient overall symptom relief**
Symptom severity^*^	Moderate	104	72.1%	96	44.8%	91	38.5%	47	29.8%
	Severe	29	48.3%	26	26.9%	22	27.3%	18	38.9%
**Number of symptoms**	≤ 4	28	78.6%	36	55.6%	23	43.5%	14	64.3%
	≥ 5	105	63.8%	86	34.9%	90	34.4%	51	23.5%
**Predominant symptom type**^**†**^	GERD	56	76.8%	36	22.2%	35	45.7%	21	23.8%
	Dyspepsia	95	68.4%	93	47.3%	77	40.3%	51	33.3%
	Other	59	62.7%	46	34.8%	55	30.9%	30	26.7%

*Moderate was defined as a GOS of 4 or 5, and severe as a GOS of 6 or 7.

^†^Symptoms were categorized as ‘GERD’ if the symptom with the highest score was heartburn or regurgitation, as ‘dyspepsia’, if the symptom with the highest score was epigastric pain or early satiety, and as ‘other’ if the symptom with the highest score was nausea/vomiting, belching or bloating. A joint highest score for more than one symptom type was possible. GERD, gastroesophageal reflux disease; GOS, Global Overall Symptom score.

## Discussion

The results of J-FOCUS, a randomized, open-label study, showed that omeprazole (10 mg once daily) was significantly more effective than famotidine (10 mg twice daily), mosapride (5 mg three times daily) or teprenone (50 mg three times daily) for providing sufficient and complete relief of upper GI symptoms after 4 weeks of treatment in endoscopically uninvestigated, *H. pylori*-negative patients. Symptoms were assessed using a previously validated symptom scale (GOS [19]), which has already been used in clinical studies to measure symptoms and treatment success in patients with dyspepsia with and without *H. pylori* infection [15,20,21]. In the present study, 66.9% of patients treated with omeprazole reached the primary endpoint of sufficient overall relief of upper GI symptoms (GOS ≤ 2) after 4 weeks of treatment, as compared with 41.0%, 36.3% and 32.3% of patients treated with famotidine, mosapride and teprenone, respectively (all, p < 0.001 vs omeprazole). The proportion of patients with complete overall symptom relief (GOS = 1) after 4 weeks of treatment was about three-fold higher in the omeprazole group (27.8%) than in the other three treatment groups (9.8%, 8.0% and 6.2%, respectively; all, p < 0.001 vs omeprazole). Within the omeprazole group, the proportion of patients with sufficient overall symptom relief after 4 weeks of treatment was highest in patients with predominant GERD symptoms, followed by those with predominant dyspepsia symptoms. None of the included patients was prescribed a PPI within 1 month of entering the study. Because PPIs are not available over-the-counter in Japan, it is very unlikely that any of the included patients had used a PPI in the month before the study.

Our results support those of 512 Canadian patients with *H. pylori*-negative dyspepsia in the randomized, double-blind CADET–*H. pylori* Negative (HN) study, which showed that omeprazole was significantly more effective than ranitidine, cisapride or placebo for relieving upper GI symptoms after 4 weeks of treatment [15]. Accordingly, both studies support the use of PPIs as first-line therapy for the treatment of dyspepsia.

Patients in J-FOCUS presented with multiple symptoms: the mean number of upper GI symptoms reported by each patient at study entry was six. The prevalence and severity of upper GI symptoms in J-FOCUS were comparable with those described in Japanese patients with GERD who had reflux esophagitis [10,23]. The mean number of upper GI symptoms per patient was 5.4 in the study by Adachi *et al.*, which included patients with GERD who had reflux esophagitis, and a similarly high symptom load was observed in the CADET-PE study, in which 80% of patients with uninvestigated dyspepsia had at least six upper GI symptoms [10,11]. We observed substantial coexistence of different symptom types, with 41.4% of patients in J-FOCUS having coexisting GERD, dyspepsia and/or other upper GI symptoms. Coexistence of GERD and dyspepsia was common, with 19.8% of patients reporting both GERD and dyspepsia as their predominant symptom types. Adachi *et al*. also observed substantial coexistence of symptoms in patients with GERD [10]. In that study, the most prevalent symptoms were heartburn and regurgitation, which were reported by 71% and 68% of patients, respectively, but these were closely followed by upper abdominal heaviness (63%), upper abdominal pain (54%) and upper abdominal fullness (53%). In our study, a lower number of upper GI symptoms at baseline was associated with treatment success, but symptom severity did not affect treatment success on logistic regression analysis.

Within each treatment group, no pronounced differences in symptom response were observed among the eight symptoms assessed by the GOS (Figure 5). Nevertheless, it cannot be ruled out that there may have been different effects on symptom subtypes consisting of epigastric pain, postprandial fullness or early satiety, as defined by the Rome III diagnostic criteria. However, we decided against conducting such analyses, because the marked coexistence of symptoms that we observed in this study would have made the data difficult to interpret, and thus, inappropriate to draw conclusions from.

The Rome III diagnostic criteria for functional dyspepsia exclude predominant symptoms of troublesome heartburn and/or regurgitation, which are diagnostic criteria for GERD, with or without reflux esophagitis [8,9]. However, dyspepsia and GERD often coexist in the same patient, and underlying GERD is a potential cause of dyspeptic symptoms [24]. GERD develops when the reflux of stomach contents causes troublesome symptoms and/or complications [8]. Acid can also play a role in dyspepsia because acid infusions into the stomach and duodenum have been shown to increase the perception of upper abdominal symptoms and affect gastroduodenal motor function in healthy individuals [25-27]. Duodenal acid exposure is increased in patients with functional dyspepsia and nausea, and this is associated with greater symptom severity [28]. Dyspepsia has also been linked with increased perception of postprandial symptoms [29], changes in visceral sensory function [30], and hypersensitivity to gastric distension [31].

GI endoscopy is useful to identify the underlying structural causes of symptoms, but can only be used to diagnose a subgroup of patients with upper GI diseases and disorders. In the CADET-PE study, the prevalence of reflux esophagitis on endoscopy was 55% (215/393) among patients with predominant heartburn and/or regurgitation, and 38% (236/647) among patients with other predominant upper GI symptoms, while only about 5% of patients had peptic ulcer disease [11]. In clinical practice in Japan, endoscopy does not usually form part of the immediate management of patients who visit their medical practitioner because of upper abdominal symptoms, unless alarm symptoms such as GI bleeding, acute weight loss or severe anemia are present. Instead, symptom control is the first priority and patients are usually treated with GI drugs with different modes of action that are selected according to the type and severity of upper GI symptoms, either in monotherapy or in combination therapy. However, up-to-date management guidelines for GERD (with and without reflux esophagitis) and dyspepsia in Japan need to be developed [16,17]. These will also need to take into account the requirement for higher doses of PPIs in view of the decreasing rate of *H. pylori* infection in Japan [32].

It is important to consider that the empirical use of PPIs for patients with uninvestigated GI symptoms should be done with care. For example, the National Institute of Health and Clinical Excellence in the United Kingdom [33] recommends that immediate referral for endoscopy (seen within 2 weeks) is indicated for progressive dysphagia, unintentional weight loss, an epigastric mass, suspicious barium meal, iron deficiency anemia or persistent vomiting. They also recommend that, in patients aged over 55 years, endoscopy should be considered if symptoms persist despite *H. pylori* testing and acid suppression therapy. Furthermore, they recommend that empirical full dose PPI therapy is offered for 1 month to patients with dyspepsia. In the present study, approximately one-third of patients treated with omeprazole and up to two-thirds of patients treated with the other drugs did not attain sufficient symptom relief. In these patients, further investigations, including endoscopy, may be necessary for diagnosis and to guide future treatments.

J-FOCUS was the first study to compare the efficacy of omeprazole, famotidine, mosapride and teprenone in Japanese patients with uninvestigated upper gastrointestinal symptoms. Its randomized, multicenter, parallel-group design further adds to the strength of this study. The present study has the following limitations: it was open label; information on over-the-counter drug use before study entry was not collected; the long-term effects of each drug beyond the 8-week trial period were not investigated; and some centers were unable to register the target number of patients meaning the allocation ratio (i.e., 2:2:2:1) was not fully met. In addition, the study did not have a placebo arm, but because we only enrolled patients with moderate to severe symptoms at inclusion made this difficult to justify from an ethical standpoint. Thus, whereas the study provided a comparison of symptom relief among active drugs with different modes of action, the therapeutic gain of each drug compared with placebo could not be determined. However, the CADET-HN study, which included a placebo arm, showed that symptom response after 4 weeks of treatment was 51% (95% CI: 43–60%) with omeprazole, 36% (95% CI: 28–39%) with ranitidine and 31% (95% CI: 22–39%) with cisapride, compared with 23% (95% CI: 16–31%) with placebo [15]. No treatment-related adverse events were reported at either follow-up visit. This may be related to the 4-week treatment duration, which was possibly too short for adverse events to occur. However, we must acknowledge the possibility that some patients who did not attend the follow-up visits may have discontinued treatment because of adverse events. It is also possible that discontinuing prior therapies 1 week before the study might have aggravated symptoms or led to the onset of new symptoms. However, we were unable to assess this possibility in the present study.

Some other limitations warrant mention. First, we used a urine antibody test to screen patients for evidence of *H. pylori* infection. Although this test shows high sensitivity and accuracy, it has not yet been recommended by guidelines as a screening test. It is therefore possible that some patients with *H. pylori* infection were included in this study. Although the use of endoscopic biopsy may have avoided this possibility, this procedure is uncomfortable for the patient, time consuming and costly. Urea breath testing, rapid urease tests or serology are well established alternatives but, considering their reported accuracies and specificities [34], they are unlikely to improve the true detection rate over that achieved with the urine antibody test in this study. Second, it is possible that the opportunity for the investigators to exclude patients based on their judgment of eligibility could have introduced some bias into the study, as the investigators may have preferentially included patients likely to respond favorably to treatment. However, we deemed this to be important and useful, as our exclusion criteria could not cover every eventuality.

## Conclusion

Our results from J-FOCUS support the implementation in Japan of European and US guidelines, which recommend PPIs as first-line treatment in patients with symptoms of GERD or dyspepsia [13,14]. Our study provides useful data for the formulation of a treatment flow algorithm in Japanese patients with upper GI symptoms, especially in view of the recent decrease in *H. pylori* infection rate among young adults in Japan [32]. Other treatment options, including different doses of PPI and combination therapy with other GI drugs, may be required in the approximately 30% of patients whose upper GI symptoms were not sufficiently relieved after 4 weeks of treatment with omeprazole. Investigation of the cause of the symptoms, such as by endoscopy, should also be considered for patients with insufficient symptom relief and in those with symptoms or clinical findings associated with high risk of serious disease or malignancy.

## Competing interests

This study was supported by grants from the Advanced Clinical Research Organization. Dr Anja Becher and Dr Christopher Winchester from Oxford PharmaGenesis provided medical writing support funded by the Japan Dyspepsia Society.

## Authors’ contributions

KS is the lead author, KH supervised the study and the other authors performed data collection. All authors have approved the manuscript.

## Pre-publication history

The pre-publication history for this paper can be accessed here:

http://www.biomedcentral.com/1471-230X/12/42/prepub
